# Anti-inflammatory therapy by ibudilast, a phosphodiesterase inhibitor, in demyelination of twitcher, a genetic demyelination model

**DOI:** 10.1186/1742-2094-2-10

**Published:** 2005-04-06

**Authors:** Kuriko Kagitani-Shimono, Ikuko Mohri, Yasushi Fujitani, Kinuko Suzuki, Keiichi Ozono, Yoshihiro Urade, Masako Taniike

**Affiliations:** 1 Department of Developmental Medicine (Pediatrics), Osaka University Graduate School of Medicine, 2-2 Yamadaoka, Suita, Osaka 565-0871, Japan; 2 Department of Molecular Behavioral Biology, Osaka Bioscience Institute, 6-2-4, Furuedai, Suita, Osaka, 565-0874, Japan; 3 Department of Pathology and Laboratory Medicine, University of North Carolina at Chapel Hill, 919 Brinkhous-Bullitt Bldg, CB7525 Chapel Hill, NC, 27599-7525, USA

## Abstract

**Background:**

Twitcher mouse (*twi/twi*) is an authentic murine model of Krabbe's disease. Accumulation of psychosine, resulting in apoptosis of oligodendrocytes and subsequent demyelination, is a cardinal event to the pathogenesis of this disease. Moreover, recruitment of inflammatory cells plays a significant role in the pathological process in the *twi/twi *central and peripheral nervous systems. In this study, we investigated the 1) the relationship between tumor necrosis factor-α (TNFα), pro-inflammatory cytokine, and the progression of this disease and 2) effect of the anti-inflammatory therapy by ibudilast, a phosphodiesterase inhibitor.

**Methods:**

We quantified the expression level of TNFα and TNF-receptor mRNA in *twi/twi *using semi-quantitative RT-PCR. The relationship between TNFα expression, apoptosis of oligodendrocytes and demyelination was studied with immunohistochemistry and TUNEL method. We then treated *twi/twi *with a daily intraperitoneal injection of ibudilast (10 mg/kg), which suppress TNFα production in the brain.

**Results:**

We found that TNFα-immunoreactive microglia/macrophages appeared in the *twi/twi *brain and that the mRNA levels of TNFα and TNF-receptor 1 was increased with the progression of demyelination. The distribution profile of TNFα-immunoreactive microglia/macrophages overlapped that of TUNEL-positive oligodendrocytes in the *twi/twi *brain. When *twi/twi *was treated with ibudilast from PND30, the number of oligodendrocytes undergoing apoptosis was markedly reduced and demyelination was milder. Obvious improvement of clinical symptom was noted in two of five. The failure of constant clinical improvement by ibudilast may result from hepatotoxicity and/or the inhibition of proliferation of NG2-positive oligodendrocyte precursors.

**Conclusion:**

We conclude that anti-inflammatory therapy by a phosphodiesterase inhibitor can be considered as a novel alternative therapy for Krabbe's disease.

## Background

The twitcher mouse (C57BL/6J-*GALC*^*twi*^; *twi/twi*) is a model of human globoid cell leukodystrophy (Krabbe's disease), a disorder caused by an inherited deficiency of the lysosomal enzyme galactosylceramidase [1-3]. *Twi/twi *shows the symptoms of cerebellar dysfunction such as action tremor and ataxia around postnatal day (PND) 25, progressive weight loss after PND 35, and cranial and peripheral nerve palsy, eventually leading to death around PND 45 [4,5]. Obvious demyelination is recognized after PND 30 in the central nervous system (CNS). Cliniconeuropathological similarities of this model and the human disease make this murine model useful for investigations of pathogenesis as well as for therapeutic approaches [6]. The pathological physiology of *twi/twi *shares many common features with that of multiple sclerosis (MS), an autoimmune demyelinating disease, including the expression of major histocompatibility complex (MHC) molecules in the CNS [7-9], activation of resident microglia, recruitment of blood-borne macrophages [10], and the strong expression of pro-inflammatory cytokines such as TNFα and interleukin (IL)-6 in the demyelinating focus [10,11]. Therefore, this murine model is useful for investigating the pathomechanism of demyelination and devising therapeutic approaches to the neuroinflammation in general.

We previously showed that demyelination of *twi/twi *was strongly associated with apoptosis of oligodendrocytes (OLs) [12]. TNFα is the most potent inducer of apoptosis of OLs among many cytokines *in vitro *[13]. Additionally, in *twi/twi *brains, TNFα was reported to be increased in demyelinating regions [11] and expression of TNFα and other immune-related molecules were down-regulated in the pathologically improved regions [10].

Phosphodiesterase inhibitors increase the intracellular cAMP levels and reduce the inflammatory cytokines such as TNFα *in vitro *[14]. Ibudilast, a non-selective phosphodiesterase inhibitor, was reported to reduce demyelination in experimental allergic encephalomyelitis (EAE) and to suppress TNFα production by microglia *in vitro *[15,16].

In this study we found that 1) the expression of TNFα and its receptor TNF-R1 was associated with demyelination and that 2) ibudilast could reduce demyelination and alleviate the progression of disease and suppress TNFα production in twitcher brain. These results were consistent with the hypothesis that TNFα signaling enhances apoptosis of OLs and demyelination in *twi/twi*, and suggested that suppression of inflammation may provide new therapeutic approaches to demyelinating diseases.

## Methods

### Animals

All animal experiments were performed according to the Guidelines for the Protection of Experimental Animals issued by the Japanese Government, the US National Institutes of Health, and the Society for Neuroscience. Heterozygous breeder pairs of twitcher (*twi/*+) were originally purchased from Jackson Laboratory (Bar Harbor, ME). *Twi/twi *and normal age-matched siblings (*+/+*) were identified by genotyping with genomic DNA extracted from the clipped tails by use of a Puregene DNA Isolation Kit (Gentra Systems, Minneapolis, MN). Genotyping was performed as previously reported [17].

### Materials

The following primary antibodies were used: phycoerythrin (PE)-conjugated anti-TNFα (1:50; PharMingen, San Diego, CA), mouse monoclonal anti-myelin basic protein (MBP) antibody (1:200; Sternberger Monoclonals Incorporated, Lutherville, MA), rabbit polyclonal anti-rat-pi-form of glutathione-S-transferase (pi-GST) antibody (1:1000; MBL, Nagoya, Japan), rabbit polyclonal anti-cow glial fibrillary acidic protein (GFAP) antibody (prediluted; DAKO, Glostrup, Denmark), biotinylated *Ricinus communis*-agglutinin-1 (RCA-1) (50 μg/ml; Vector Laboratories, Burlingame, CA), and rabbit polyclonal NG2 chondroitin sulfate proteoglycan (NG2) antibody (1:200; Chemicon International Inc., Temecula, CA). Biotinylated *Ricinus communis*-agglutinin-1 (RCA-1) (50 μg/ml) was purchased from Vector Laboratories (Burlingane, CA).

### Tissue preparation

Brains from *twi/twi *and *+/+* mice killed at PND 20, 30, and 40 (n = 3 for each timing period) were immunostained for TNFα. The mice were perfused with cold physiological saline under deep inhalation anesthesia with sevoflurane, and the isolated brains were quickly frozen in liquid nitrogen. For routine histochemical staining, mice (n = 3 for each groups) were perfused with physiological saline, followed by 4% paraformaldehyde in 0.1 M phosphate buffer (PB, pH 7.4). The brain was removed, postfixed and embedded in paraffin blocks. Luxol fast blue (LFB)-periodic acid Schiff (PAS) staining was performed on the paraffin sections of *twi/twi *and *+/+* at PND 40 for evaluation of neuropathology.

For the determination of mRNA levels, groups of *twi/twi *and *+/+* (n = 3 each timing period) were killed at PND 20, 30, and 40 under appropriate anesthesia. The brains were then removed, divided into the cerebrum and cerebellum/brain stem, and quickly frozen in liquid nitrogen.

### Immunocytochemistry

Frozen sections were fixed at 4°C in acetone and incubated with PE-conjugated rat anti-mouse TNFα antibody for 48 h. For double labeling with RCA-1 and anti-TNFα, TNFα-stained sections were reacted with biotinylated RCA-1 for 30 min at room temperature, and then with avidin-D-fluorescein isothiocyanate isomer (avidin-FITC; Vector Laboratories), diluted 1:1000 with PBS, for 30 min. For NG2 immunostaining, after blocking with 0.3% Triton-X100 for 1 h, frozen sections were incubated with anti-NG2 antibody for 12 h at 4°C, and incubated with Alexa 488-conjugated anti-rabbit IgG (H+L) (1:400; Molecular Probes, Inc., Eugene, OR) for 2 h.

Paraffin sections were used for immunostaining for MBP and pi-GST, and terminal deoxynucleotidyltransferase (TdT)-mediated dUTP nick end labeling (TUNEL). For immunocytochemistry, sections on glass slides were incubated serially with mouse anti-MBP or rabbit anti-pi-GST antibody, biotinylated goat anti-mouse or anti-rabbit immunoglobulins (Vector Laboratories), and avidin-biotin complex by using an ABC elite kit (ABC; Vector Laboratories). Immunoreactions were visualized by immersing the slides in a 0.03% H_2_O_2 _solution in 50 mM Tris-HCl (pH 7.6) containing 0.05% diaminobenzidine tetrahydrochloride (DAB) and 0.25% nickel ammonium sulfate. *Twi/twi *and *+/+* at PND 40 were subjected to TUNEL staining. Nuclei with DNA fragmentation were detected by using an *in situ *apoptosis detection kit (Takara Biomedicals, Osaka, Japan). Briefly, after pretreatment with 0.1% trypsin for 15 min at 37°C, sections were reacted with TdT, dNTPs, and FITC-labeled dUTP for 90 min at 37°C, followed by horseradish peroxidase (HRP)-conjugated anti-FITC antibody overnight at 4°C. The immunoproduct was visualized with the same protocol described above.

To identify the type of TUNEL-positive cells, we combined the staining for pi-GST, GFAP and RCA-1 with the TUNEL procedure. After TUNEL staining, sections were incubated with PBS containing 0.3% TritonX-100 and 10% normal goat serum for 30 min and then with rabbit anti rat-pi GST antibody, rabbit anti-cow GFAP antibody or biotinylated RCA-1 at 4°C overnight. The procedures were basically the same as described above except for the use of ABC-alkaline phosphatase and naphthol AS-BI phosphate coupled with hexazotized new fuchsin (Merck, Darmstadt, Germany) as a chromogen.

### Quantification of the level of TNFα-mRNA

Total RNA was isolated from the quick-frozen brains with Isogen (Nippon gene, Toyama, Japan). The random 9-mers-primed cDNA was prepared with an RNA-LA-PCR Kit (Takara Shuzo, Kyoto, Japan) and 2 μg of total RNA.

A LightCycler PCR and detection system (Roche Diagnosis, Mannheim, Germany) was used for the amplification and quantification of mRNA for TNFα, TNFR1, TNFR2 and glycerol aldehyde-3-phosphate dehydrogenase (G3PDH) as previously described [18]. G3PDH served as an internal control. The sequence-specific primers used were as follow: TNFα forward primer: 5'-AGTGACAAGCCTGTAGCCCACG-3', TNFα reverse primer: 5'-TTTCTCCTGGTATGAGATAGC-3', TNFR1 forward primer: 5'-CTAAACAGCAGAACCGAGTGT-3', TNFR1 reverse primer: 5'-AGATACGTAGAGTGTCCTTGG-3', TNFR2 forward primer: 5'-ATAAAGCCACACCCACAACCT-3', TNFR2 reverse primer: 5'-CATCTCCCTGCCACTCACAA-3', G3PDH forward primer: 5'-TGAACGGGAAGCTCACTGG-3', and G3PDH reverse primer: 5'-TCCACCACCCTGTTGCTGTA-3'. The constructs, used to create a standard curve, were made by cloning each amplified fragment into the Hind III site of a pGEM vector (Promega, Madison, WI). The number of copies was calculated by plotting a dilution series on this standard curve in each PCR experiment. For amplification detection, the LightCycler DNA Master Hybridization Probes Kit was used. Quantification of TNFα mRNA was performed by conducting 50 cycles of repeated denaturation (1 s at 89°C), annealing (5 s at 58°C), and enzymatic chain extension (10 s at 72°C). The PCR amplification conditions for G3PDH were 40 cycles of repeated denaturation (1 s at 87°C), annealing (5 s at 57°C), and enzymatic chain extension (10 s at 72°C). Quantification of TNFR1 and TNFR2 mRNAs was made by using 50 cycles of repeated denaturation (1 s at 89°C), annealing (5 s at 58°C), and enzymatic chain extension (10 s at 72°C). Duplicated PCR products were evaluated by melting curve analysis.

### Administration of Ibudilast

Ibudilast was a generous gift from Kyorin Pharmaceutical Co. Ltd. (Tokyo, Japan). After dissolved to a concentration of 1 mg/ml in physiological saline containing 10% v/v of polyoxyethylene hydrogenated castor oil 60 (HCO60), ibudilast (10 mg/kg) was injected intraperitoneally daily into three *twi/twi *from PND 15 to PND 40, and five *twi/twi *from PND 30 to PND 45. For controls, the same volume of HCO 60 was injected into two *twi/twi *from PND 15 to PND 40 and four *twi/twi *from PND 30 to PND 45. The density of TUNEL-positive cells in the demyelinating lesion in *twi/twi*, treated from PND 30 to PND 45 was calculated by using MacSCOPE software (Mitani Co, Fukui, Japan). Two independent neuropathologists examined the LFB-PAS-stained coronal sections (four sections per mouse) at the level of the optic chiasm and at the cerebellopontine angles containing the paraflocculus in a double-blind manner and scored the severity of demyelination from 0 to 5. 0: no demyelination, 1: slight demyelination, 2: less than 25% of the areas are occupied by a demyelination focus, 3: 25% ~ 50% of the areas occupied, 4: 50 ~ 75% of the areas occupied, 5: more than 75% of the areas occupied. The scores were average of two examiners' evaluations.

### In situ hybridization for TNFα

The cDNA probe for TNFα comprised a 268-bp PCR fragment (forward primer; 5'-GATGGGTTGTACCTTGTCTACTCC-3' and reverse primer; 5'-CTAAGTACTTGGGCAGATTGACCT-3') from the mouse TNFα, and was subcloned into a pGEM-T Easy vector (Promega, Madison, Wisconsin). *In situ *hybridization was carried out by using manual capillary action technology with a Microprobe staining system (Fisher Scientific International, Hampton, NH) as previously described [19,20]. First, brain sections (10 μm) were deparaffinized with Auto Dewaxer (Research Genetics, Huntsville, AL). The sections were rinsed in Auto Alcohol, Universal Buffer, and Immuno/DNA buffer (Research Genetics). Predigestion by proteinase K (15 μg/ml; Sigma-Aldrich, St Louis, MO) was performed to increase the tissue penetration of the probe. After this digestion, the tissue sections were treated with Immuno/DNA buffer. The DIG-labeled cRNA probe was diluted to 0.5 μg/ml with Brigati probe diluent (Research Genetics), 50% deionized formamide, and 50% dextran sulfate. The probe solution was heated at 90°C to denature the cRNA structures and applied to the slides. The hybridization of tissue and probe was done at 50°C for three hours. After hybridization, the slides were washed in 2 × SSC containing nonionic detergent. The detection of the DIG-labeled RNA was performed by using the Genius DNA labeling and detection kit (Roche Diagnostics). For counterstaining, neutral red was applied.

### Statistical analysis

Student's t test was performed by using Stat View software (SAS Institute, Cary, NC). p< 0.05 was considered as significant.

## Results

### Levels of TNFα and TNFR1 are increased in the twitcher cerebellum

**Figure 1:**
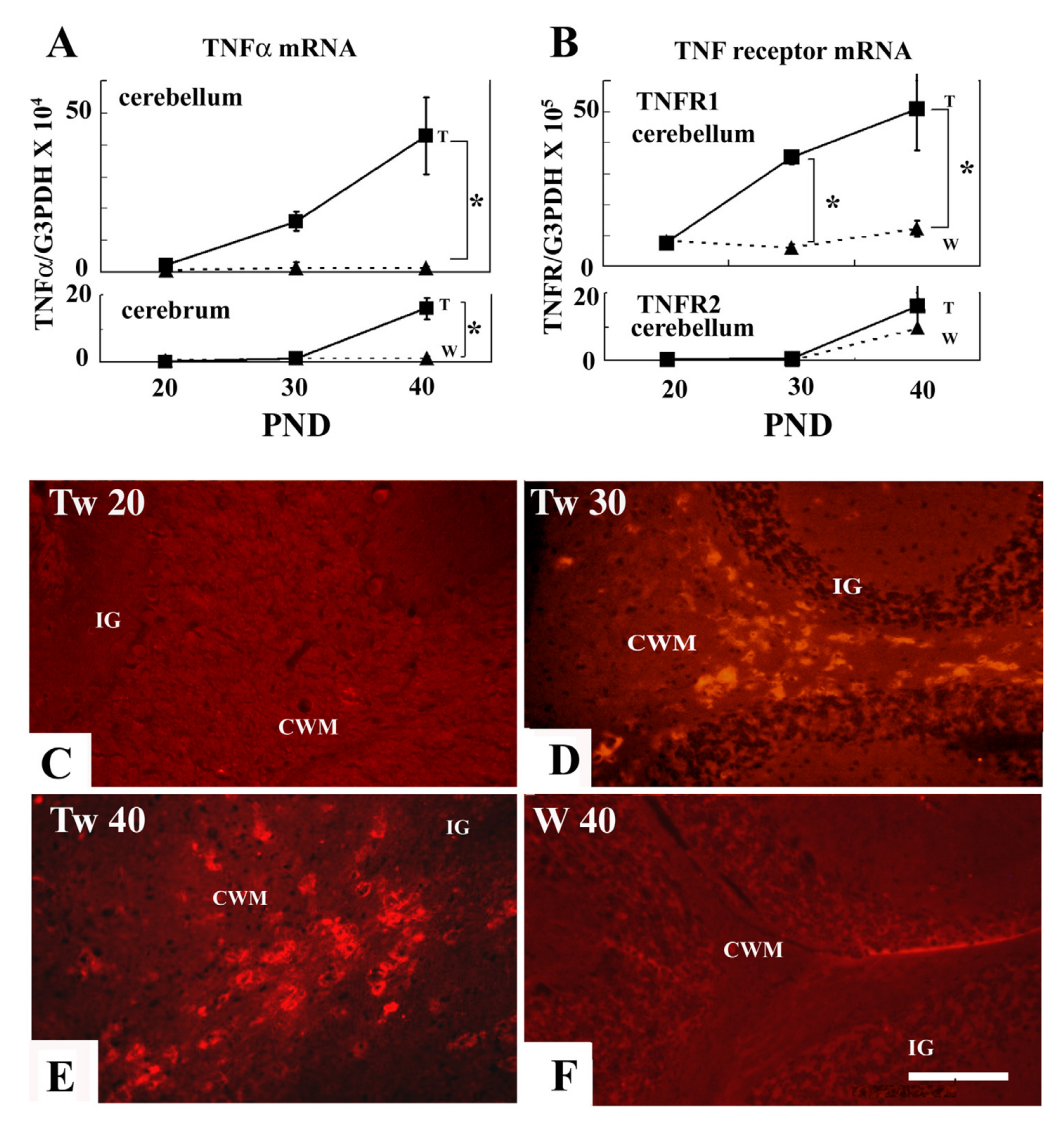
TNFα and its receptors increased as demyelination proceeded. A-B: Quantification of mRNA for TNFα (A) and its receptors (B). The copies of mRNA for TNFα have increased in *twi/twi *(■) after PND 30, especially in the cerebellum, when compared with those in +/+ (▴). Those for TNFR1 in the cerebellum have increased in *twi/twi *after PND 30. The copies of mRNA for TNFR2 have increased in *twi/twi *only after PND 40, when compared with those for +/+, but the difference was not significant (B). Bar represents mean ± SE. * p < 0.01. C-F: TNFα immunostaining in the cerebellum. There are no TNFα-positive cells in the cerebellum of *twi/twi *mice at PND 20 (C). Immunoreactive cells for TNFα are progressively increased in number in the *twi/twi *cerebellar white matter between PND 30 (D) and PND 40 (E). In contrast, there are no TNFα positive cells in +/+ brains at any ages examined (F). Tw and W represent *twi/twi *and wild-type mice, respectively. The data represent mean ± SE. IG: internal granular layer, CWM: cerebellar white matter. Scale bar = 50 μm.

The level of TNFα mRNA was the same in both cerebellum and cerebrum of the *+/+* at any age examined. In the cerebrum, the level of TNFα-mRNA in *twi/twi *was almost the same as that in *+/+* until PND 30, however, it increased to become approximately 15 times higher at PND 40 than that of *+/+*. In the cerebellum, there was no difference in the TNFα mRNA level between *twi/twi *and *+/+* at PND 20, however, its level increased significantly in *twi/twi *after PND 30, becoming 40 times higher in *twi/twi *than *+/+* at PND 40 (Fig. 1A).

Next, we investigated the levels of TNFR1 and TNFR2. In the *+/+* cerebellum, the level of TNFR1 mRNA was constant throughout all the ages examined, whereas in the *twi/twi *cerebellum, it significantly increased with the progression of demyelination, becoming 50 times higher than that in *+/+* at PND 40. In contrast, mRNA for TNFR2 increased in *twi/twi *only after PND 40, when compared with that for *+/+* (Fig. 1B).

Immunocytochemical analysis revealed that TNFα-immunoreactive cells were not recognized at PND 20 (Fig. 1C) in *twi/twi*. However, many TNFα-immunoreactive cells were found in the cerebral white matter, brain stem and cerebellar white matter (CWM) at PND 30 (Fig. 1D) and 40 (Fig. 1E). On the other hand, TNFα-immunoreactive cells were not detected anywhere in the *+/+*brain even at PND 40 (Fig. 1F). These data were compatible with the data of the quantitative RT-PCR.

### TNFα expression is increased in microglia/macrophages within demyelinating lesions in *twi/twi*

**Figure 2:**
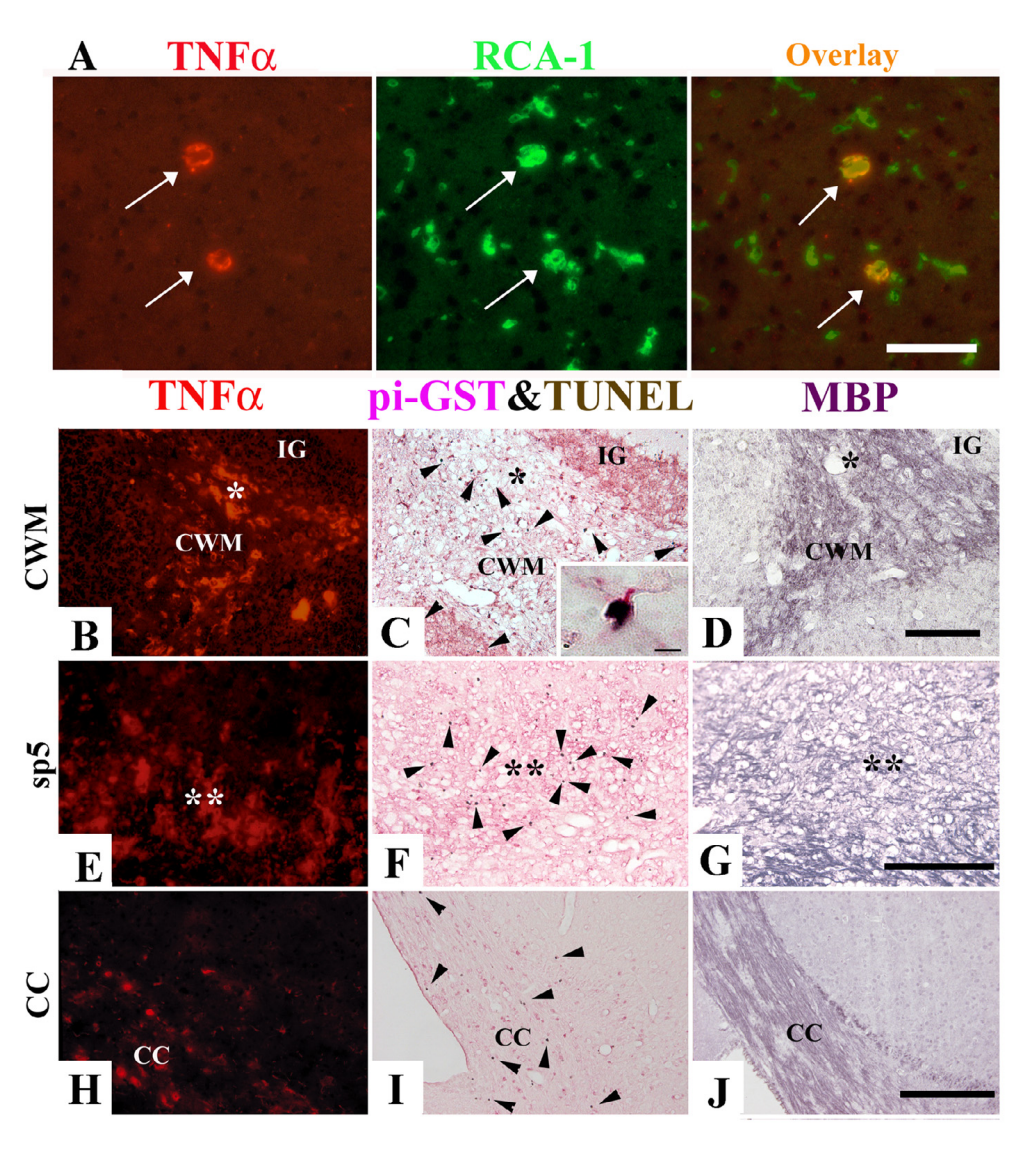
TNFα is expressed in activated microglia/macrophages in the regions where many apoptotic OLs are recognized with severe demyelination. A: Double labeling of TNFα and RCA-1 of the *twi/twi *cerebrum at PND 40. Arrows indicate microglia/macrophages, which are double positive for TNFα and RCA-1. B-J : In *twi/twi *at PND 40, there are many TNFα-positive cells (B, E) as well as many TUNEL-positive cells (C, F) in the CWM and sp5, where severe demyelination was present as judged from the results of MBP immunostaining (D, G). These apoptotic cells are immunostained with pi-GST, identified to be OLs (inset in C). In the corpus callosum (cc), there are only a few TNFα-positive cells (H) and TUNEL-positive cells (I), where demyelination was milder than in the cerebellum (J). Asterisks and double asterisks represent the same region in the serial sections. Scale bars = 50 μm (B-J), 10 μm (inset in "C").

The morphological characteristics of TNFα-positive cells were an irregular cellular contour and lack of delicate processes, reminiscent of ameboid microglia/macrophages. Furthermore, TNFα-positive cells were positive for RCA-1, a marker for macrophage (arrows in Fig. 2A), but negative for pi-GST, a marker for OLs, or GFAP, a marker for astrocytes (data not shown), confirming those cells to be microglia/macrophages. In the *twi/twi *brain, both TNFα-positive cells and TUNEL-positive cells were most abundant in the CWM (Fig. 2B, C) and in the spinal trigeminal tract (sp5) in the superior midbrain (Fig. 2E, F). The majority of TUNEL-positive cells were also positive for pi-GST (arrowheads in Fig. 2C, F, I), identifying them as OLs (inset in Fig. 2C). These lesions of the cerebellum were most severely demyelinated judged by MBP immunostaining (Fig. 2D, G). In contrast, in the corpus callosum, where demyelination was milder than in the cerebellum, only a few TNFα-positive cells were detected (Fig. 2H – J).

### Administration of phosphodiesterase inhibitor ameliorates demyelination and the clinical symptoms

**Figure 3:**
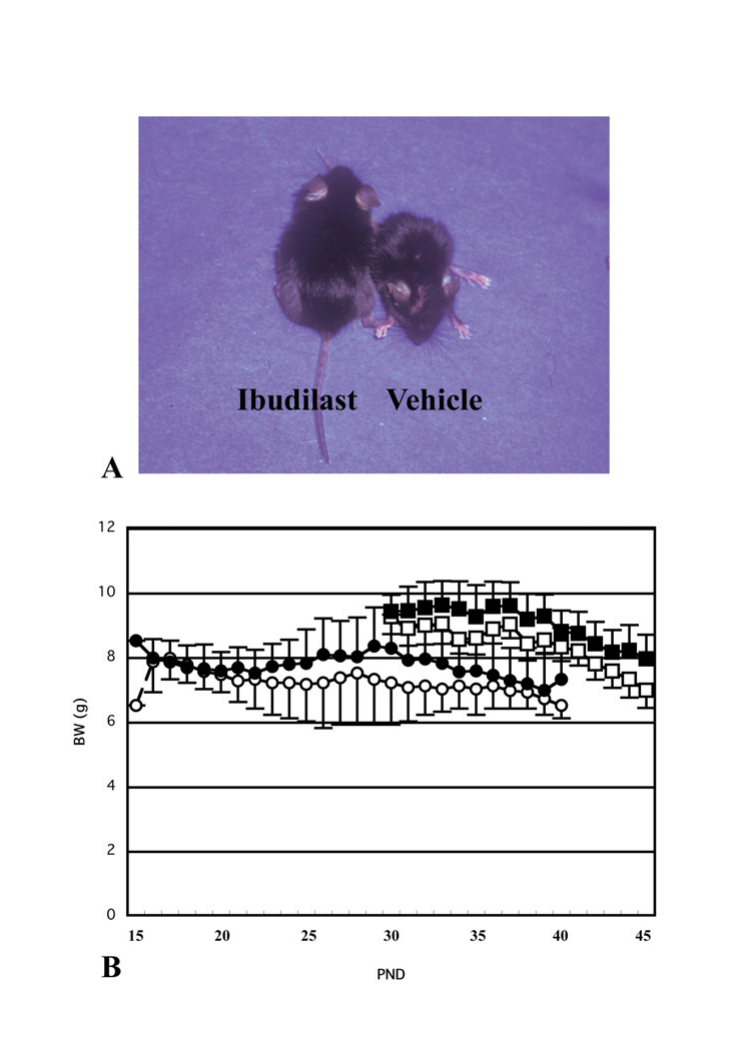
**A**: Two *twi/twi *at PND 44, one ibudilast-treated and other vehicle-treated from PND 30. The ibudilast-treated *twi/twi *is much bigger and can walk faster and reach the feedbox, in spite of mild paralysis and spasticity in lower limbs. In contrast, the vehicle-treated *twi/twi *can no longer walk nor feed itself. In addition, the ibudilast-treated *twi/twi *has much milder tremor than the vehicle-treated *twi/twi*. **B**: The change of body weight (g) of ibudilast- and vehicle-treated *twi/twi*. Both *twi/twi *treated with ibudilast or vehicle from PND 15 (●: ibudilast-treated *twi/twi*, ○: vehicle-treated *twi/twi*) showed less weight gain compared with those treated from PND 30 (■: ibudilast-treated *twi/twi*, □: vehicle-treated *twi/twi*), and no prolongation of the life span. However, ibudilast-treated *twi/twi *showed less body weight loss than vehicle-treated *twi/twi*. N = 3 and 2 in ibudilast- and vehicle-treated *twi/twi *from PND 15. The ibudilast-treated *twi/twi *from PND 30 were bigger and showed milder clinical detrerioration. N = 5 and 4 in ibudilast- and vehicle-treated *twi/twi *from PND 30. The data represent mean ± SE.

To investigate whether the inflammatory response in microglia/macrophages contributes to the demyelination in *twi/twi*, we administered a phosphodiesterase inhibitor, ibudilast, to *twi/twi*. Two out of five *twi/twi *treated from PND 30 revealed strikingly milder clinical symptoms (Fig. 3A). Even at PND 45, two of ibudilast-treated *twi/twi *from PND 30 could move smoothly despite mild hindlimb paralysis, and showed less severe tremor and ataxia than vehicle-treated *twi/twi*. These mice were bigger than vehicle-treated *twi/twi*, as they had less weight loss (Fig. 3B). In contrast, ibudilast-treated *twi/twi *from PND 15 showed neither apparent clinical improvement nor elongation of lifespan, however, their body weights were heavier than those of vehicle-treated *twi/twi*.

**Figure 4:**
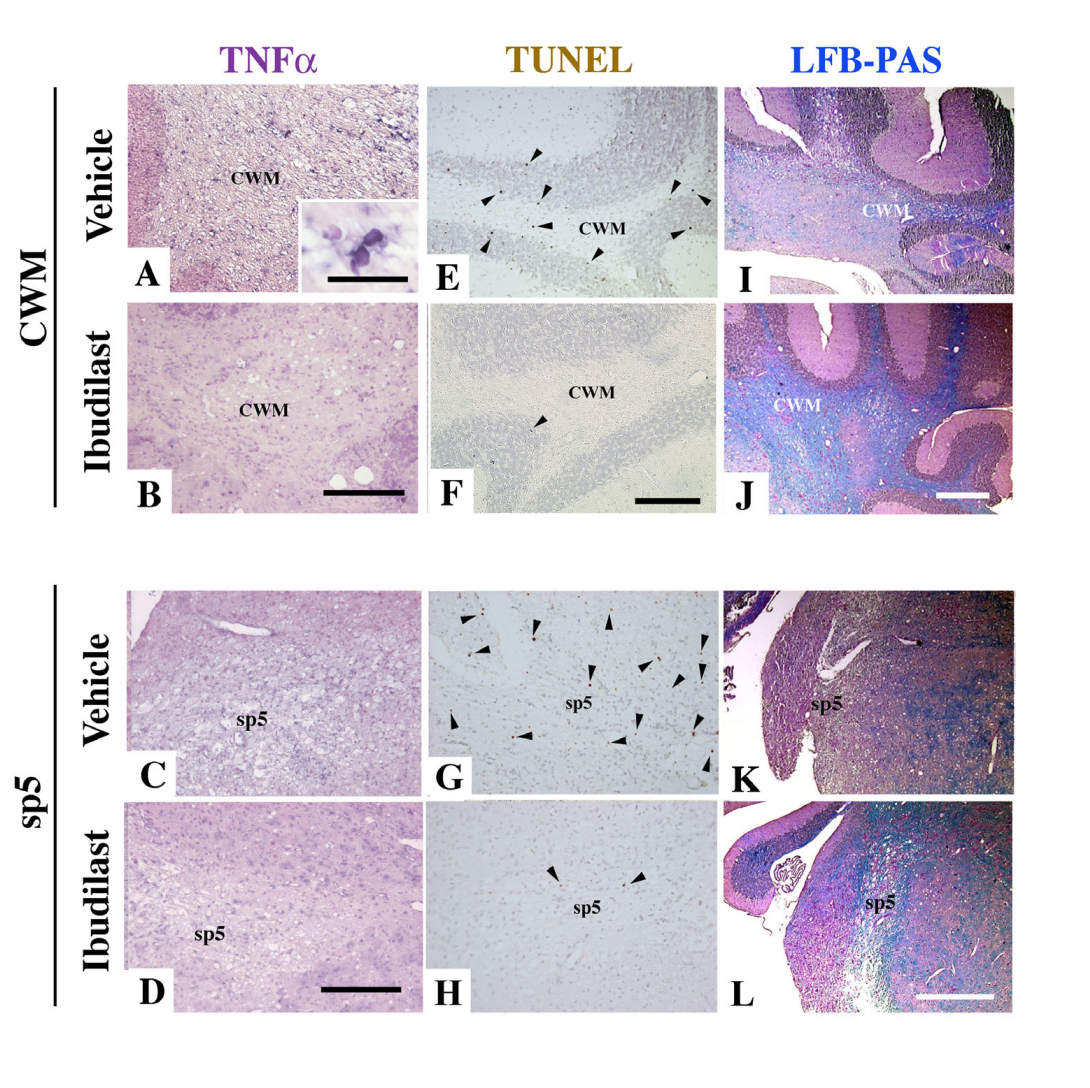
Suppression of TNF mRNA expression is accompanied by inhibition of apoptosis and subsequent milder demyelination in ibudilast-treated *twi/twi *at PND45. A, B, E, F, I, J: CWM, C, D, G, H, K, L: sp5. A-D: *In situ *hybridization of TNFα mRNA in vehicle-treated *twi/twi *(A, C) and ibudilast-treated *twi/twi *(B, D). Whereas vehicle-treated *twi/twi *show abundant signals in CWM (A) and sp5 (C), TNFα mRNA signals are remarkably reduced in the ibudilast-treated *twi/twi *(B, D). Inset in "A" shows TNF-α mRNA-positive microglia. E-H: TUNEL staining of vehicle-treated *twi/twi *(E, G) and ibudilast-treated *twi/twi *(F, H). Ibudilast-treated *twi/twi *shows fewer TUNEL-positive cells than are seen in vehicle-treated *twi/twi*. Arrowheads indicate TUNEL-positive cells. I-L: LFB-PAS staining of vehicle-treated *twi/twi *(I, K) and ibudilast-treated *twi/twi *(J, L). In the ibudilast-treated *twi/twi*, CWM and sp5 show much milder demyelination than in vehicle-treated *twi/twi*. Scale bar = 100 μm (I-L), 50 μm (A-H), 10 μm (inset in "A").

**Figure 5:**
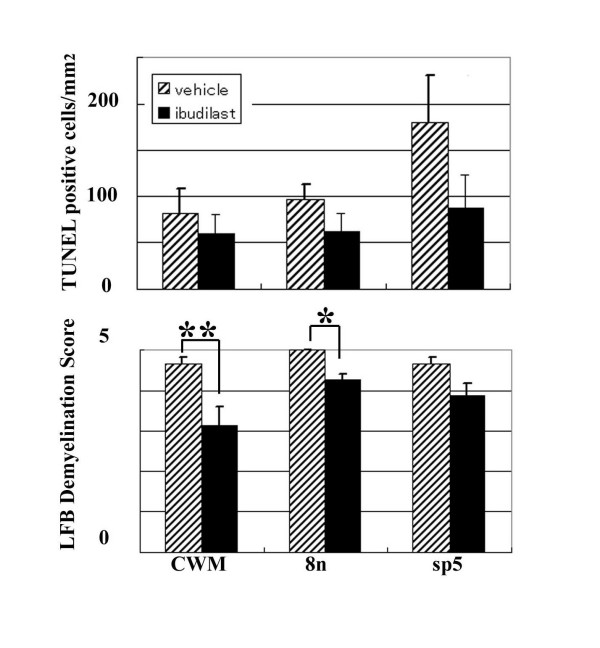
Ibudilast-treated *twi/twi *show pathological improvement. Population of TUNEL-positive cells and neuropathological scores of LFB-PAS in ibudilast- (closed-boxed; N = 4) or vehicle-treated (hatched; N = 3) *twi/twi*. In CWM, 8 n, and sp5 of the ibudilast-treated *twi/twi*, the number of TUNEL-positive cells is decreased to half of those in the vehicle-treated *twi/twi*. They also recognized significantly milder demyelination in LFB-PAS stain. 8 n: the 8^th ^nerve. *p < 0.01, **p < 0.05. The error bars represented standard deviations.

The signal for TNFα mRNA obtained by *in situ *hybridization was recognized in the cells with small nuclei in the CWM and sp5 of vehicle-treated *twi/twi *(inset in Fig. 4A), corresponding to the presence of TNFα-immunoreactivity in the microglia. This signal was significantly reduced in the ibudilast-treated *twi/twi *(Fig. 4B, D). The number of TUNEL-positive cells was decreased in the CWM in ibudilast-treated *twi/twi *(Fig. 4F, H) compared with that of the vehicle-treated mice (Fig. 4E, G). TUNEL-positive cells were decreased in other regions such as the 8^th ^nerve (8 n) and sp5 in ibudilast-treated *twi/twi *than in vehicle-treated mice (Fig. 5, the upper bar graph).

LFB-PAS staining revealed that the demyelination was remarkably suppressed in the ibudilast-treated mice from PND 30 (Fig. 4J, L) compared with the vehicle-treated ones (Fig. 4I, K), as shown in the score of demyelination (Fig. 5, lower bar graph). From these lines of evidence, we concluded that the demyelination and clinical symptoms were reduced with inhibition of TNFα in *twi/twi*.

#### Ibudilast treatment decreased NG2-positive OL progenitors

**Figure 6:**
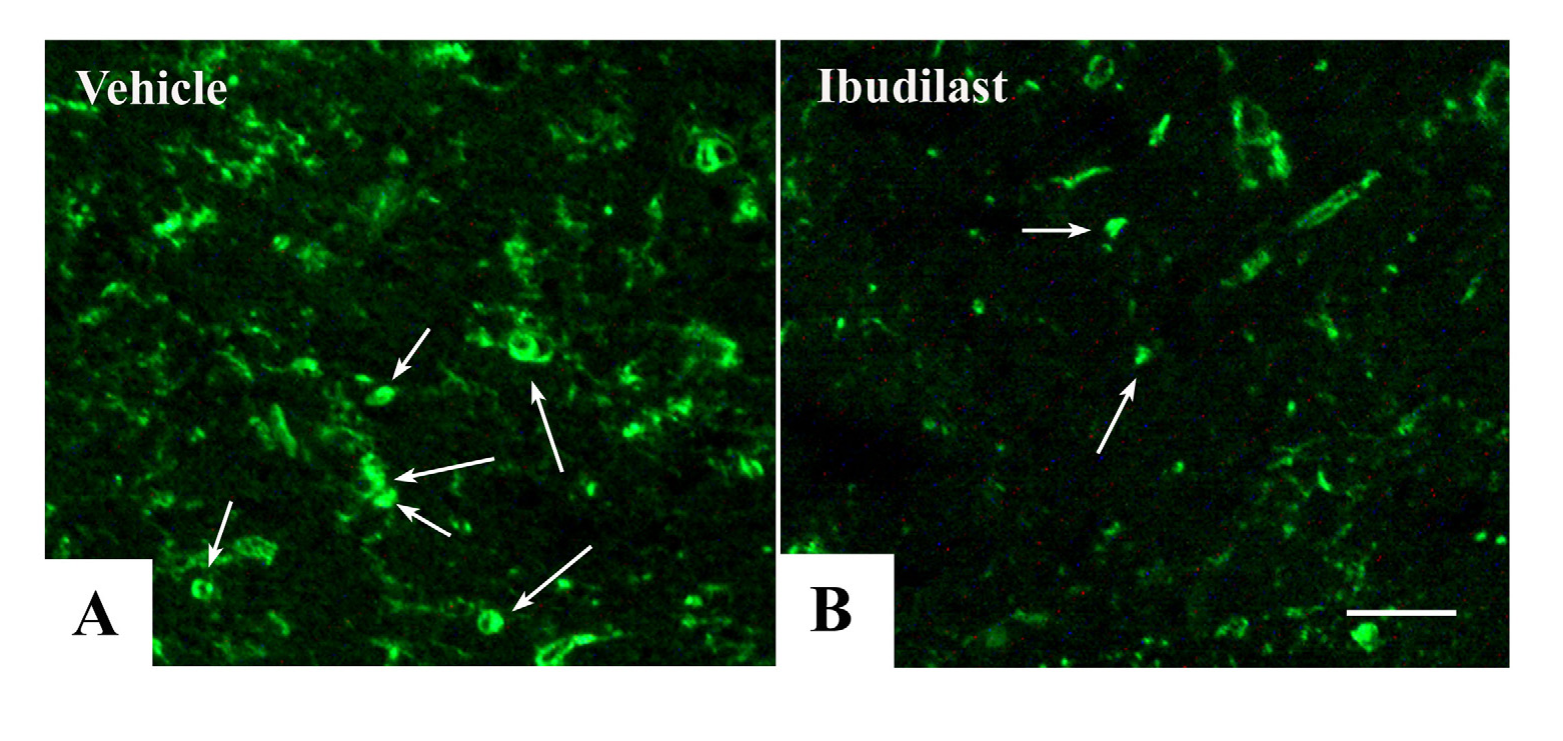
Ibudilast surpresses proliferation of NG2-positive OL progenitors. A: Vehicle-treated *twi/twi *shows many NG2-positive OL progenitors. B: Ibudilast-treated *twi/twi *shows decreased number of NG2-positive OL progenitors. Allows: NG2-positive OL progenitors labeled with Alexa 488. Scale bar = 50 μm

To evaluate the effect of ibudilast to the OL progenitors, frozen sections were stained with anti-NG2 antibody. In contrast to the vehicle-treated *twi/twi*, ibudilast-treated *twi/twi *showed fewer NG2-positive OL progenitors (Fig. 6), suggesting that incomplete clinical improvement may result from the insufficient remyelination in ibudilast-treated *twi/twi*.

## Discussion

Our results suggested that secondary inflammation via TNFα produced in microglia/macrophages remarkably enhances the apoptosis of OLs and aggravates the demyelination due to the metabolic defect in *twi/twi*. These are consistent with previous reports showing that TNFα induces apoptosis of OLs *in vitro *[21,22], and that TNFα is upregulated in macrophages and globoid cells in *twi/twi *[11].

TNFα is a well-established pro-inflammatory mediator of immune process, and is essential to the maintenance of CNS homeostasis. However, its overexpression leads to the development of chronic CNS inflammation and degeneration [23]. We previously observed emergence of TNFα-expressing cells with progression of demyelination and the number of those cells declined following bone marrow transplantation with prolonged survival in *twi/twi *[10]. TNFα was expressed by infiltrating blood mononuclear cells, and its expression was well correlated with the extent of demyelination in another genetic demyelinating disease, X-linked adrenoleukodystrophy[24], and in the MS [25]. TNFα-transgenic mice showed more severe demyelination and macrophage infiltration in EAE, a mouse model for MS [26]. Of two TNFRs, TNFR1 was reported to mediate the pathogenetic effects of TNFα, such as inflammation, cytotoxicity, and apoptosis of OLs in EAE [13,27-29]. Our study showed that TNFR1 was dominant from the early demyelinating stage and that demyelination and OL apoptosis was alleviated by the suppression of TNFα in ibudilast-treated *twi/twi*. These lines of evidence suggested that the stimulation of TNFR1 was associated with apoptosis of OLs and demyelination in *twi/twi*. Therefore, we believe that TNFα/TNFR1-mediated secondary inflammation is involved in the progression of pathology in varieties of demyelinating diseases.

In this study, we selected ibudilast as an immunomodulatory agent which also suppressed the production of other inflammatory mediators, such as nitric oxide (NO), IFN-γ, and IL-6, and enhanced the production of the inhibitory cytokine, IL-10, and neurotrophic factors, including nerve growth factor (NGF), glia-derived neurotrophic factor (GDNF) and neurotrophin (NT-4) [30]. Since inducible nitric oxide (iNOS) and IL-6 were strongly upregulated in *twi/twi *and Krabbe's disease [10,11,31], the positive effect of ibudilast may be also associated with suppression of iNOS and IL-6, and enhancement of inhibitory cytokines and neurotrophic factors. However, taking into account that TNFα is the most potent cytotoxic cytokine, and that signals for TNFα mRNA were remarkably suppressed in the areas of severe demyelination in ibudilast-treated *twi/twi*, the effect of ibudilast may be mediated, at leaset in part, by the suppression of TNFα expression.

Several different types of anti-TNFα therapy have been recently reported. For example, TNF-receptor-p55-immunoglobulin fusion protein was reported to suppress demyelination in EAE [32,33], whereas it showed no significant efficacy in MS patients [34,35]. Infliximab and etanercept, used as anti-TNFα agents for rheumatoid arthritis and Crohn's disease, are rather reported to induce demyelination [36,37]. In contrast to the poor outcomes of these direct TNFα suppression, interferon (IFN) β [38,39] and glatiramer acetate (GA) [40,41] have been widely approved as effective immunomodulatory treatments for MS. TNFα production was significantly reduced in monocytes from patients treated by GA [42], which acts primarily as an antigen for T lymphocytes. Furthermore, MS patients who received administration of IFNβ revealed decreased mRNA for TNFα [43] and an increase in serum TNFRs, of which TNFR2 may play a protective role for myelin [44].

The clinical symptoms were improved in only two ibudilast-treated *twi/twi*, whereas the demyelination was milder in all of the treated *twi/twi*. In the ibudilast-treated *twi/twi *without clinical improvement, the number of NG2-immunoreactive OL progenitors was decreased, compared with that in vehicle-treated *twi/twi*. Lack of TNFα has been reported to result in a significant delay of remyelination in a cuprizone-induced demyelination model, due to a reduced number of proliferating OL progenitors [45], since the signal transduction of TNFα via p75 TNF receptor 2 (TNFR2) is known to induce proliferation of OL progenitors [27,28]. Therefore, TNFα stimulation may be involved not only in the apoptotic signal pathway mediated by TNFR1, but may also play a regenerative role via activation of TNFR2 [46]. Earlier treatment with ibudilast from PND 15 showed less apparent clinical effect compared with that from PND30, probably due to the following two reasons: daily intraperitoneal injection itself could be too invasive for younger *twi/twi *to gain weight and/or TNFR2-stimulated proliferation of OLs in this period of active myelination is profoundly inhibited by the reduced TNFα production. These lines of evidence suggested that TNFα inhibitor should be used for a limited period of time or in a TNFR1-specific manner.

The cytotoxicity of ibudilast may be another explanation for the failure of clinical improvement in some cases: when we administered a high dosage (20 mg/kg) of ibudilast to *twi/twi*, it induced vacuolar degeneration of hepatocytes and the mice died of the hepatic failure (data not shown). When ibudilast was directly administered by an intraventricular injection to avoid systemic adverse effect, periventricular tissues were extensively damaged by this chemical. These results indicate that other drugs with less cytotoxicity are necessary to improve the symptoms of *twi/twi *and other demyelination diseases.

From these lines of evidence, we propose that anti-inflammatory therapy by a phosphodiesterase inhibitor during an appropriate period, may be a reliable supportive treatment for Krabbe's disease for which there is no effective treatment except bone marrow transplantation [6,23,47-49].

## Conclusion

These results suggest that the suppression of inflammation by a phosphodiesterase inhibitor could be a novel therapy in genetic demyelination.

## List of abbreviations

twitcher mouse (*twi/twi*)

tumor necrosis factor-α (TNFα)

postnatal day (PND)

central nervous system (CNS)

multiple sclerosis (MS)

major histocompatibility complex (MHC)

interleukin (IL)

oligodendrocytes (OLs)

experimental allergic encephalomyelitis (EAE)

phycoerythrin (PE)

myelin basic protein (MBP)

pi-form of glutathione-S-transferase (pi-GST)

glial fibrillary acidic protein (GFAP)

*Ricinus communis*-agglutinin-1 (RCA-1)

phosphate buffer (PB)

fluorescein isothiocyanate isomer (FITC)

terminal deoxynucleotidyltransferase (TdT)-mediated dUTP nick end labeling (TUNEL)

diaminobenzidine tetrahydrochloride (DAB)

horseradish peroxidase (HRP)

Luxol fast blue (LFB)-periodic acid Schiff (PAS)

glycerol aldehyde-3-phosphate dehydrogenase (G3PDH)

cerebellar white matter (CWM)

interferon (IFN)

glatiramer acetate (GA)

nitric oxide (NO)

nerve growth factor (NGF)

glia-derived neurotrophic factor (GDNF)

neurotrophin (NT)

inducible nitric oxide synthase (iNOS)

## Competing interests

The author(s) declare that they have no competing interests.

## Authors' contributions

KKS was responsible for the majority of the experimental studies, and for writing the manuscript. IM and YF contributed to technical tutorship and the editing of the manuscript. KS and KO contributed to editing of the manuscript. MT and YU contributed to the conception, interpretation of results and the writing and editing of the manuscript. All authors read and approved the final manuscript.
